# Supplementary material for: Unsupervised vector-based classification of single-molecule charge transport data
Source: Nat Commun. 2016 Oct 3;7:12922. doi: 10.1038/ncomms12922 (PMC5063956; doi:10.1038/ncomms12922)
Supplement: Supplementary Information — Supplementary Figures 1-23, Supplementary Notes 1-4 and Supplementary References [file ncomms12922-s1.pdf]

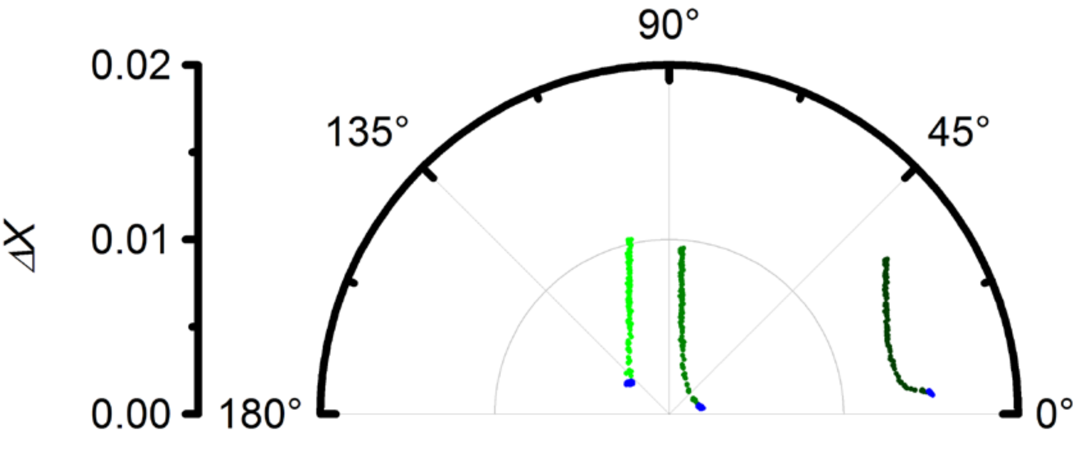

Supplementary Figure 1 - Variation of Reference Vector: Three times the same simulated data set (RoI: 0.2 nm to 2 nm, s_b 0.9 nm, s_b STDEV 1 nA, Noise 0.1 nA, I_p 1nA, plateau containing graphs in green and plain exponential graphs in blue). Reference vectors 0.5 Å^{-1} 1 Å^{-1} and 5 Å^{-1} (dark to light green). Changing the reference vector does not fundamentally alter the distribution. Plain exponentially decaying graphs are at the bottom of the distribution for all three reference vectors. However, a “good” choice of the reference vector can aid the cluster assignment step.

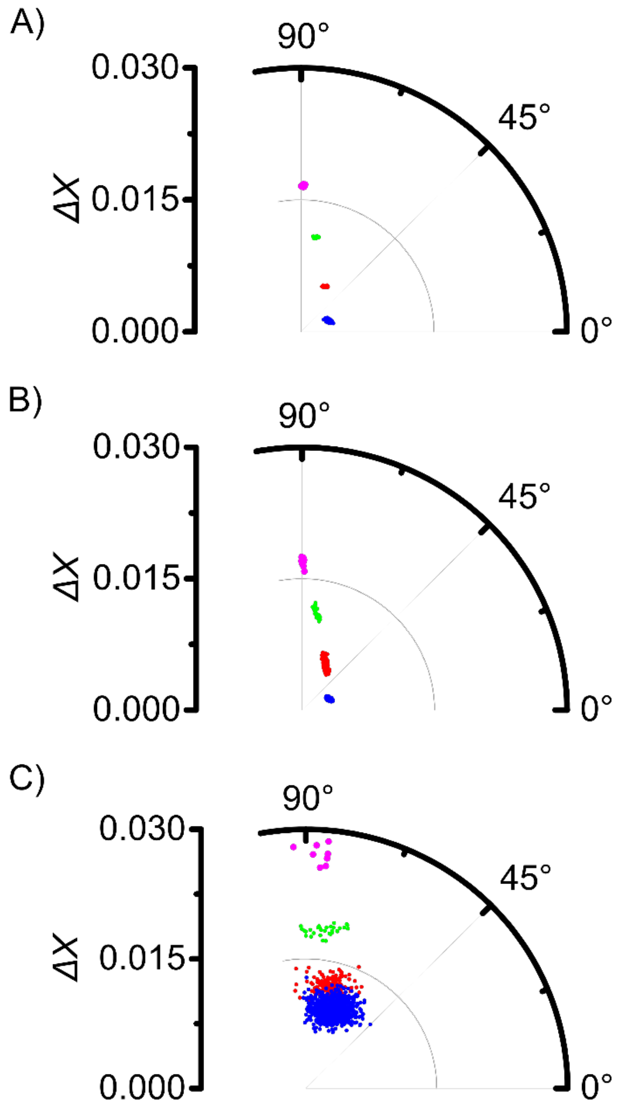

Supplementary Figure 2 - 1, 2 or 3 molecules in the junction: Cylinder plot viewed along the z-axis of a simulated data set, mimicking a junction consisting of 1, 2 or 3 molecules. The probability decreases with the number of molecules in the junction. A) Noise STDEV 0.1 nA. B) Noise STDEV 0.1 nA, I_p STDEV 0.1 nA. C) Noise STDEV 1 nA. (RoI: 0.1 nm to 2 nm)

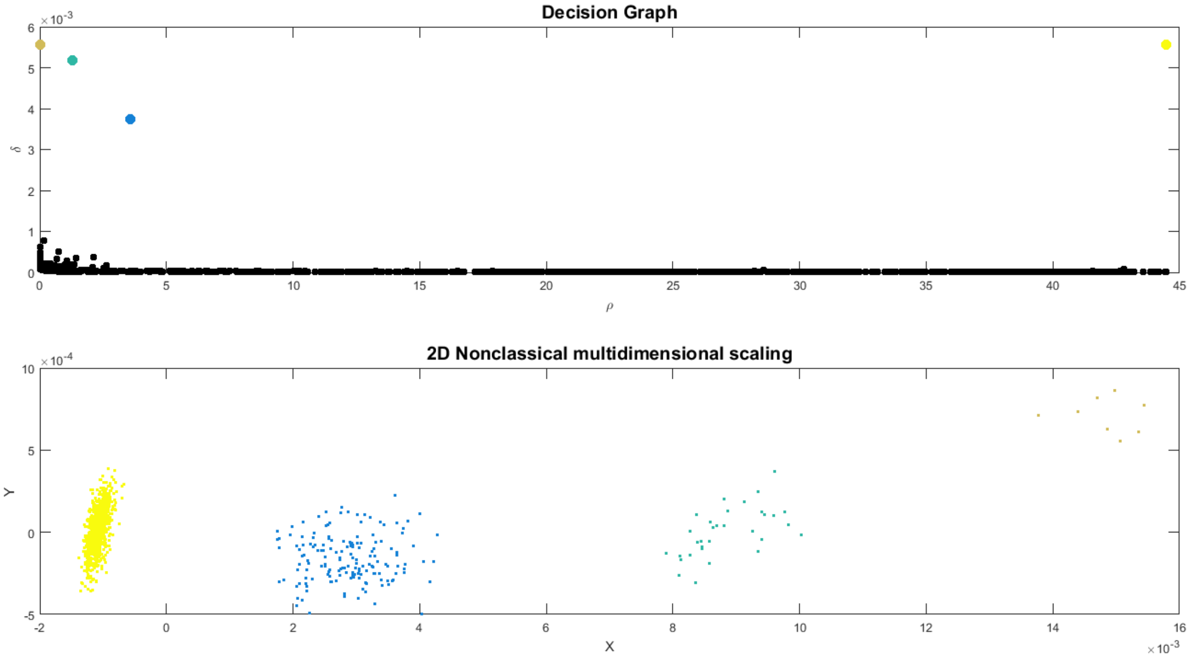

Supplementary Figure 3- Density Clustering: Data set from Supplementary Figure 2 B), analysed with density clustering[1]. Decision graph (top) and clustering assignment (bottom). Clustering was performed adapting the algorithm provided by Rodriguez and Laio.

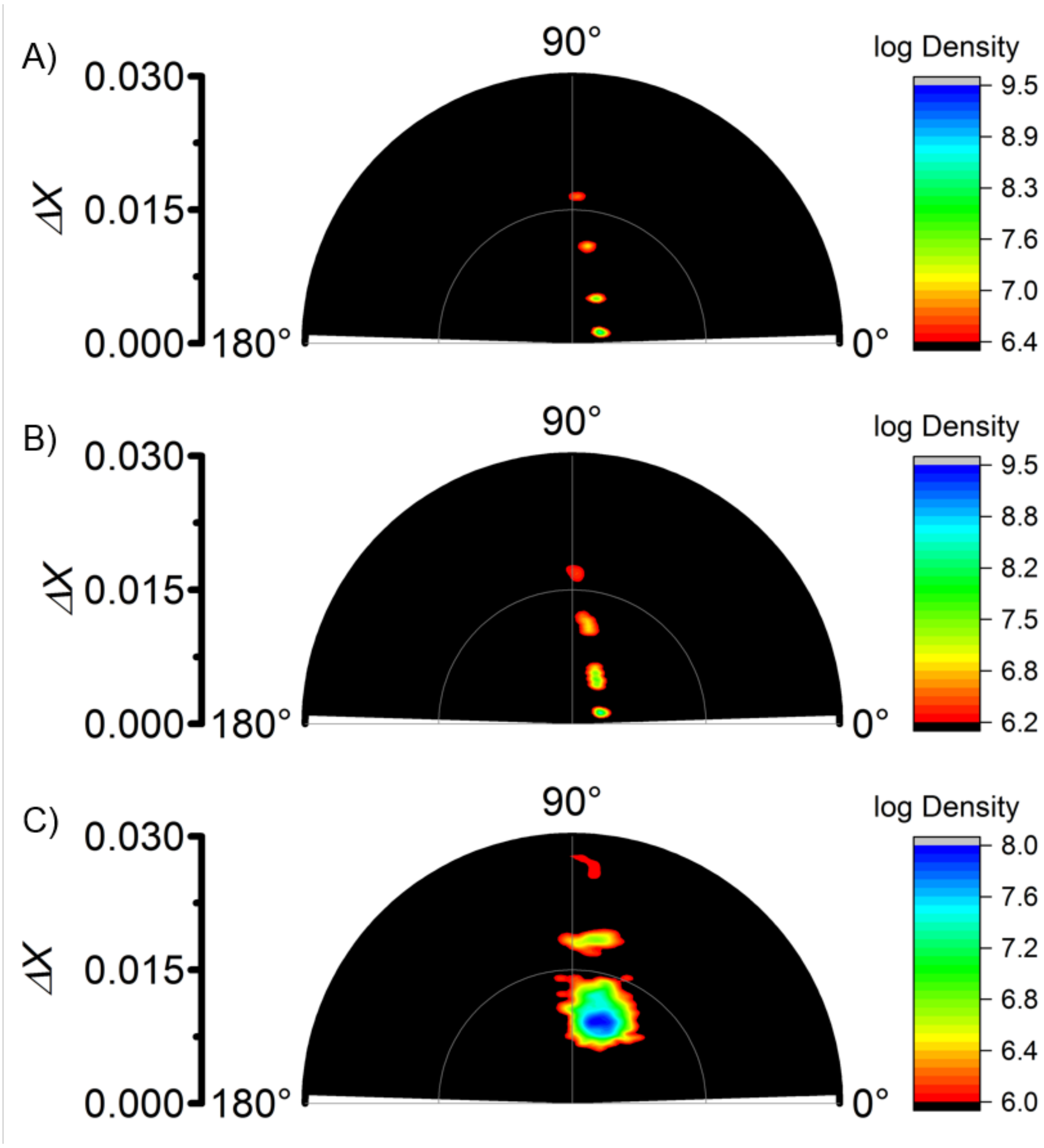

Supplementary Figure 4 - Point density of 1, 2 or 3 molecules in the junction: 50 radial bins/0.03 and 50 angular bins/180°. Data sets as in Supplementary Figure 2. The number of clusters used in the clustering step can be rationalized by plotting the point density. Areas of high point density indicate clusters in the data.

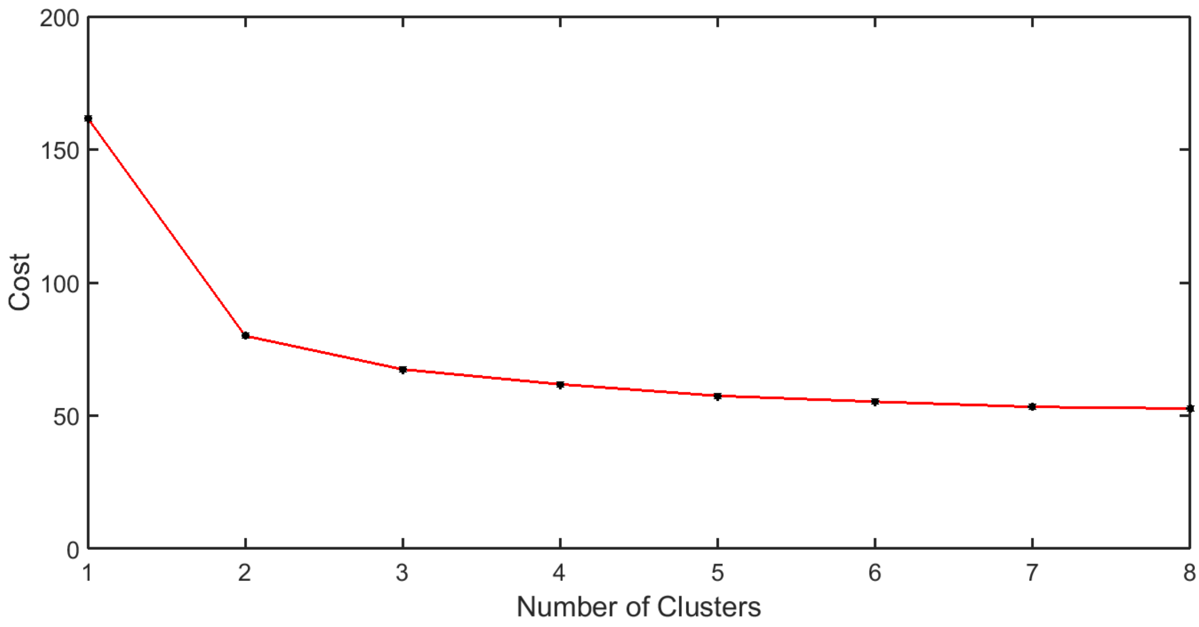

Supplementary Figure 5 - Cost function versus the number of clusters for OPE: Clustering algorithms aim to minimize a cost function associated with the total residual distance that all observations have to their cluster centres. There is usually no exact solution for the number of clusters with unlabelled data. Plotting the cost versus the number of clusters can give an indication of the optimal number of clusters ("elbow" in the graph).

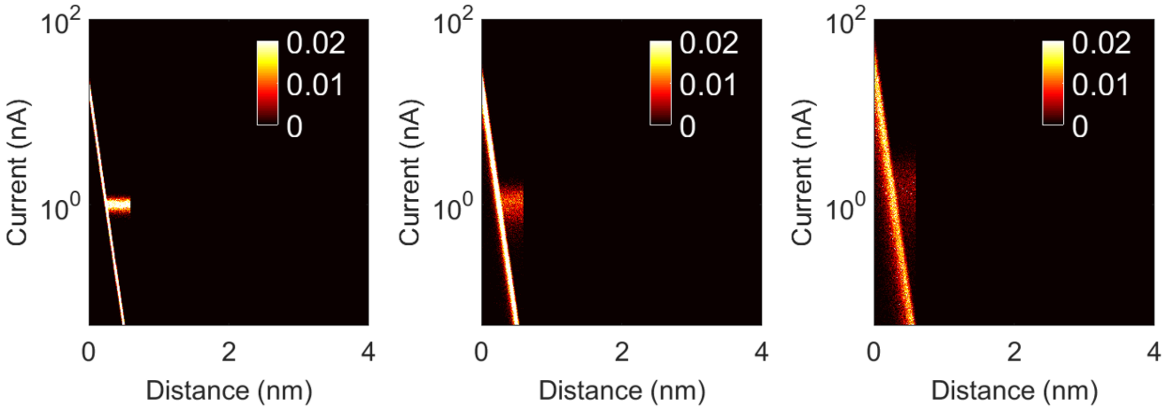

Supplementary Figure 6 - Noise: 2D log current histograms of the data sets used in Figure 2 of the main text with noise STDEV 0.1 nA, 0.3 nA and 1 nA (left to right). The clear plateau at 0.1 nA STDEV diminishes with increasing noise. At 1 nA noise STDEV, the S/N is very low, making it difficult to assign a plateau current to the data. (Current binning: 150 bins/decade, distance bin width: 0.004 nm for all 2D histograms)

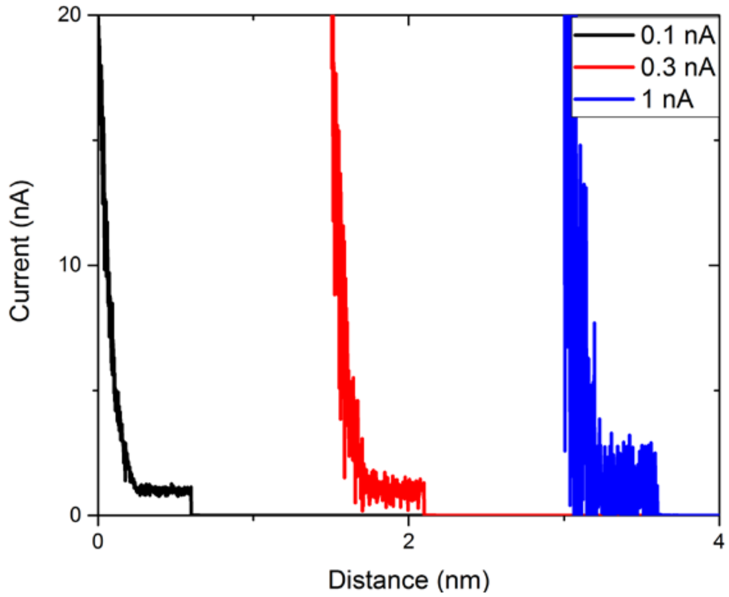

Supplementary Figure 7 - Noise: Individual *I(s)* traces with the different noise levels used in Figure 2 of the main text. Sample Graphs with noise STDEV 0.1 nA, 0.3 nA and 1 nA (left to right, offset for clarity)

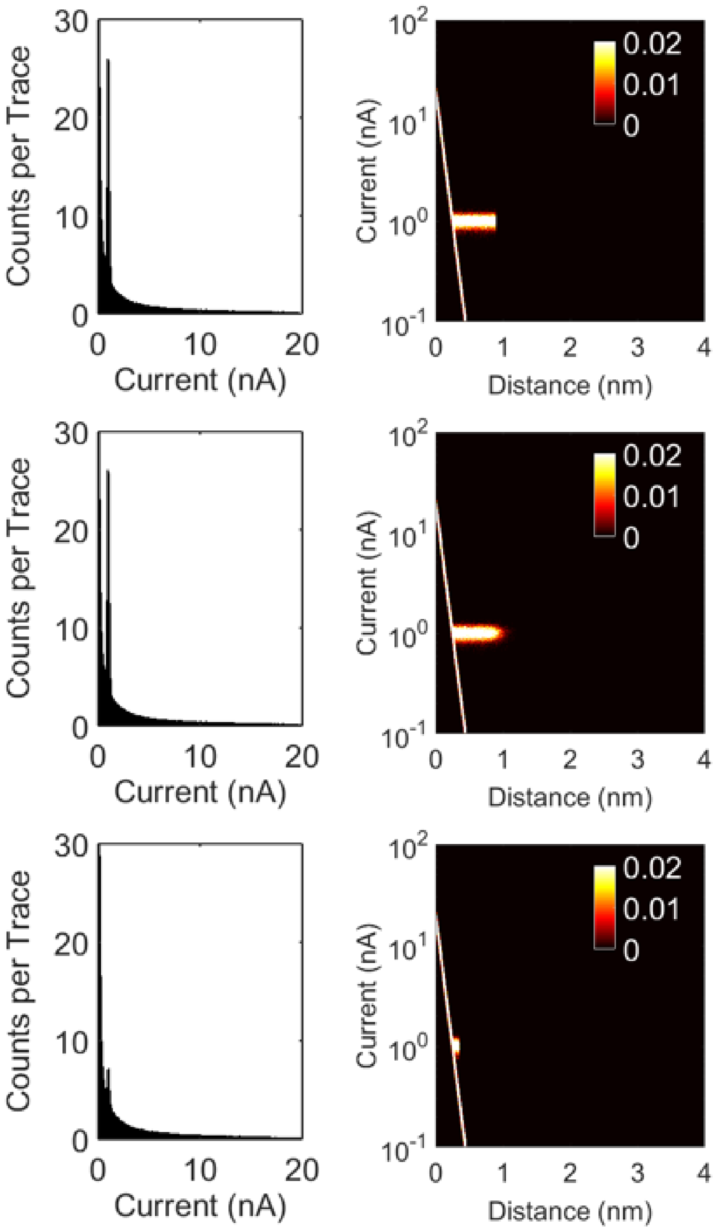

Supplementary Figure 8 - Histograms at variations of break-off distance: Data set used in Figure 3 of the main text. s_b of 0.9 nm, 0.9 nm with STDEV 0.1 nm and 0.35 nm (Top to bottom). 1D current and 2D log current histograms. The clear current peaks at long s_b disappear with decreasing plateau length. (Current binning: 150 bins/decade, distance bin width: 0.004 nm for all 2D histograms, 1D current histogram bin width: 0.1 nA)

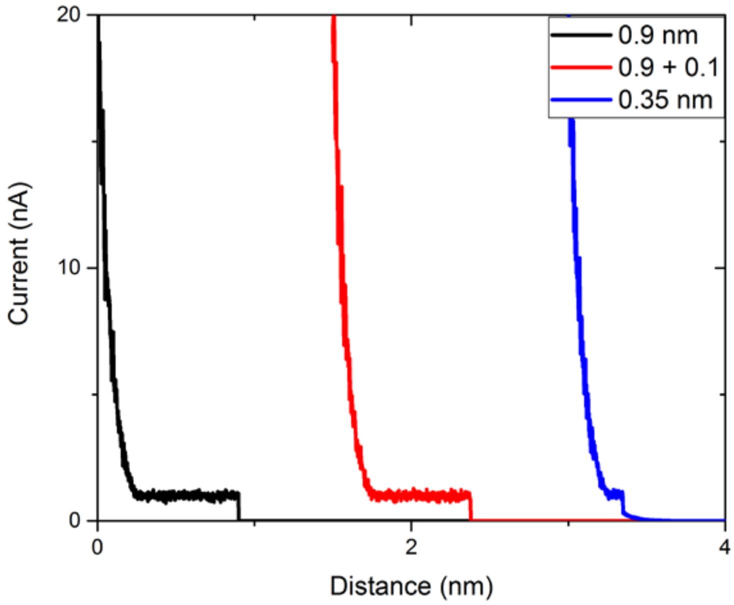

Supplementary Figure 9 - Variation of Break-off - *I(s)* traces: s_b 0.9 nm, random *I(s)* trace from the cluster of *I(s)* traces with s_b 0.9 nm and STDEV of 0.1 nm and 0.35 nm (Left to right, offset for clarity)

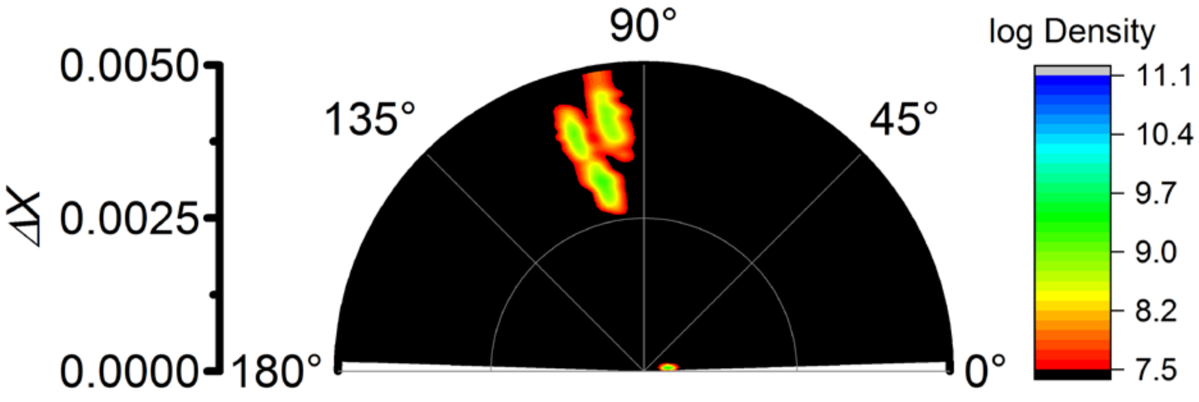

Supplementary Figure 10 – simulated data Point density of data set with different plateau shapes: Logarithmic point density of data set used in Figure 4 of the manuscript with three different plateau shapes and plain exponential *I(s)* traces.

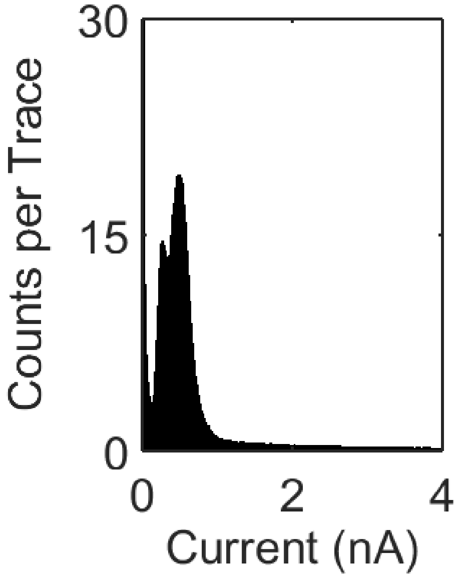

Supplementary Figure 11 – simulated data - plateau shapes: All data 1D current histogram of the data used in Figure 4 of the manuscript, containing three different plateau shapes and plain exponentially decaying *I(s)* traces. Different peaks can be seen in this representation, but three different types of plateaus cannot be identified. (Bin Width: 0.02 nA)

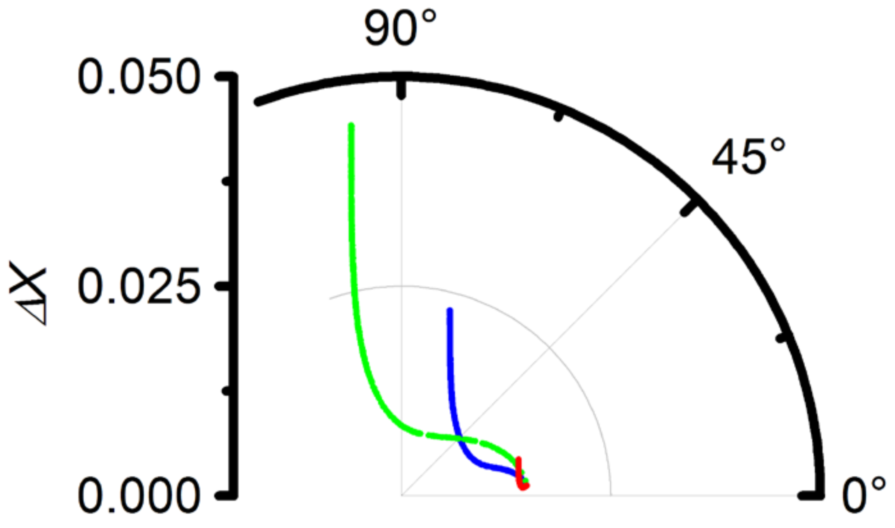

Supplementary Figure 12- simulated data - limitations: 1000 graphs each with I_p 0.5 nA (red) 2.5 nA (blue) and 5 nA (green) with s_b STDEV 1 nm (RoI: 0.2 nm to 2 nm), Reference β: 0.5 $Å^{-1}$. Overlapping and elongated clusters are difficult to assign with clustering algorithms like FCM.

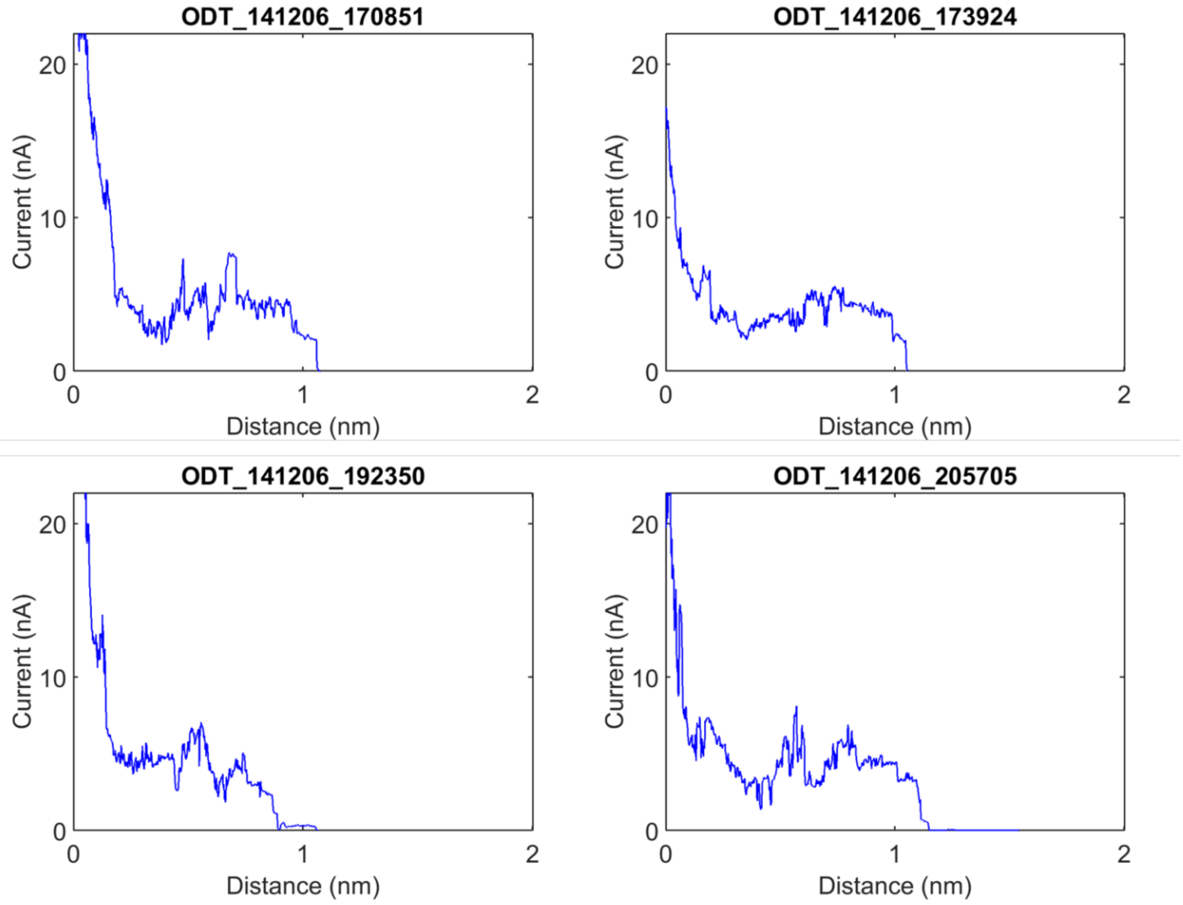

Supplementary Figure 13 – ODT – high *G*: Representative *I(s)* traces of the high conductance region.

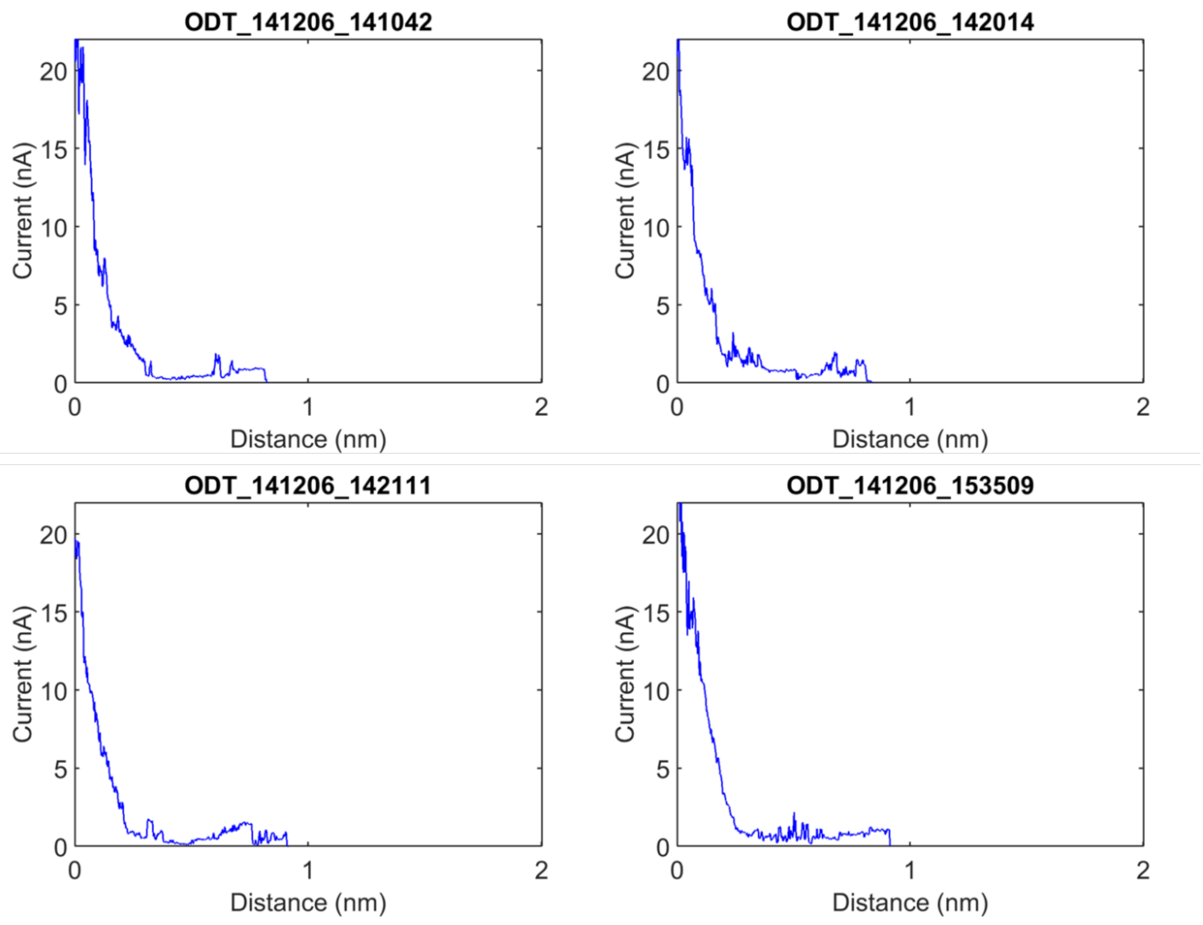

Supplementary Figure 14 – ODT – low *G*: Representative *I(s)* traces of the low conductance region.

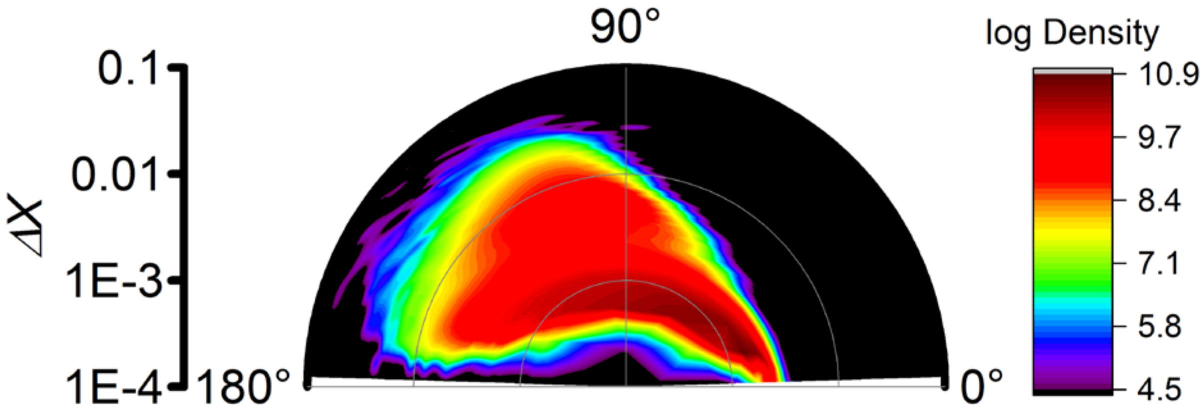

Supplementary Figure 15 - ODT: Logarithmic point density plot of experimental ODT data (Note the radial logarithmic scale). The semi-circular cluster at the origin of the plot contains plain exponential traces. The cluster towards larger *ΔX* contains traces with plateaus. This representation does not indicate any sub-clustering in the molecular cluster.

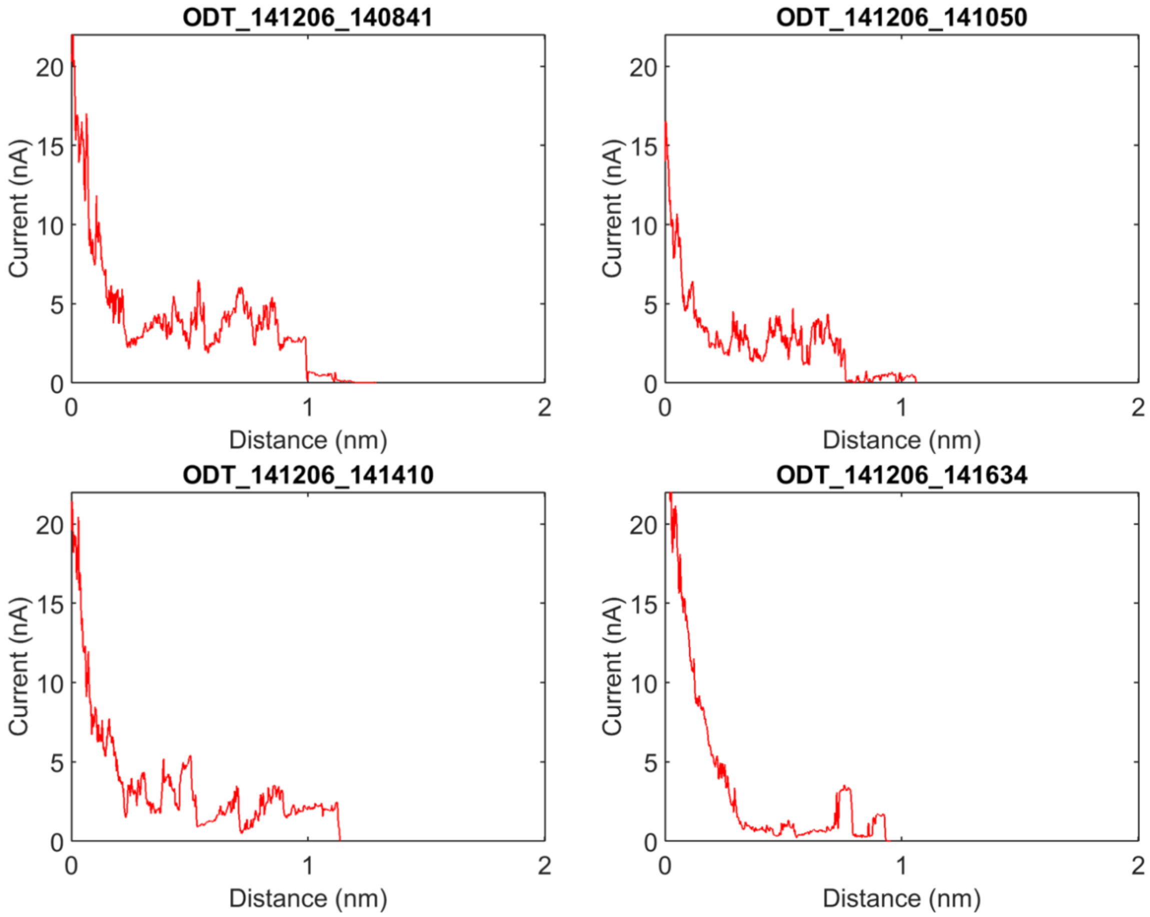

Supplementary Figure 16 – ODT - Red cluster: Selection of representative traces of the red cluster. Containing traces switching between high, medium and low conductance state.

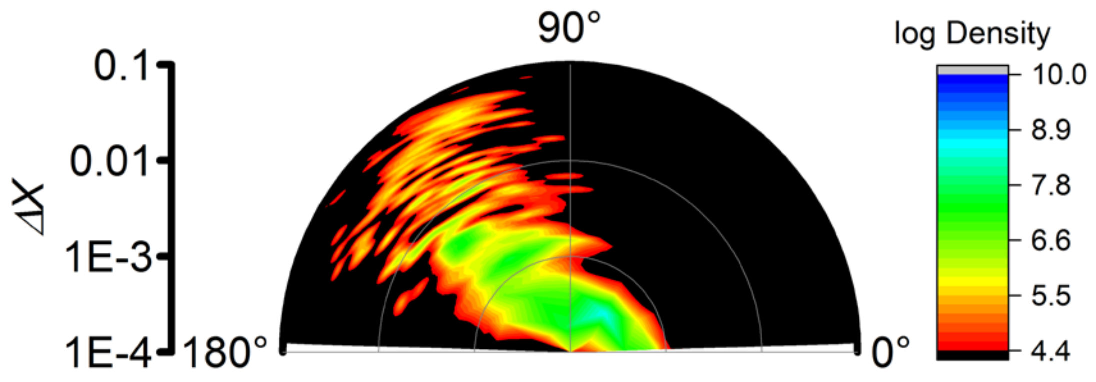

Supplementary Figure 17 - Point Density - OPE: Logarithmic point density of experimental OPE data set. (15 bins per decade in radial direction, 50 angular bins). Three different clusters can be seen, corresponding to plain exponential, low conductance and high conductance cluster.

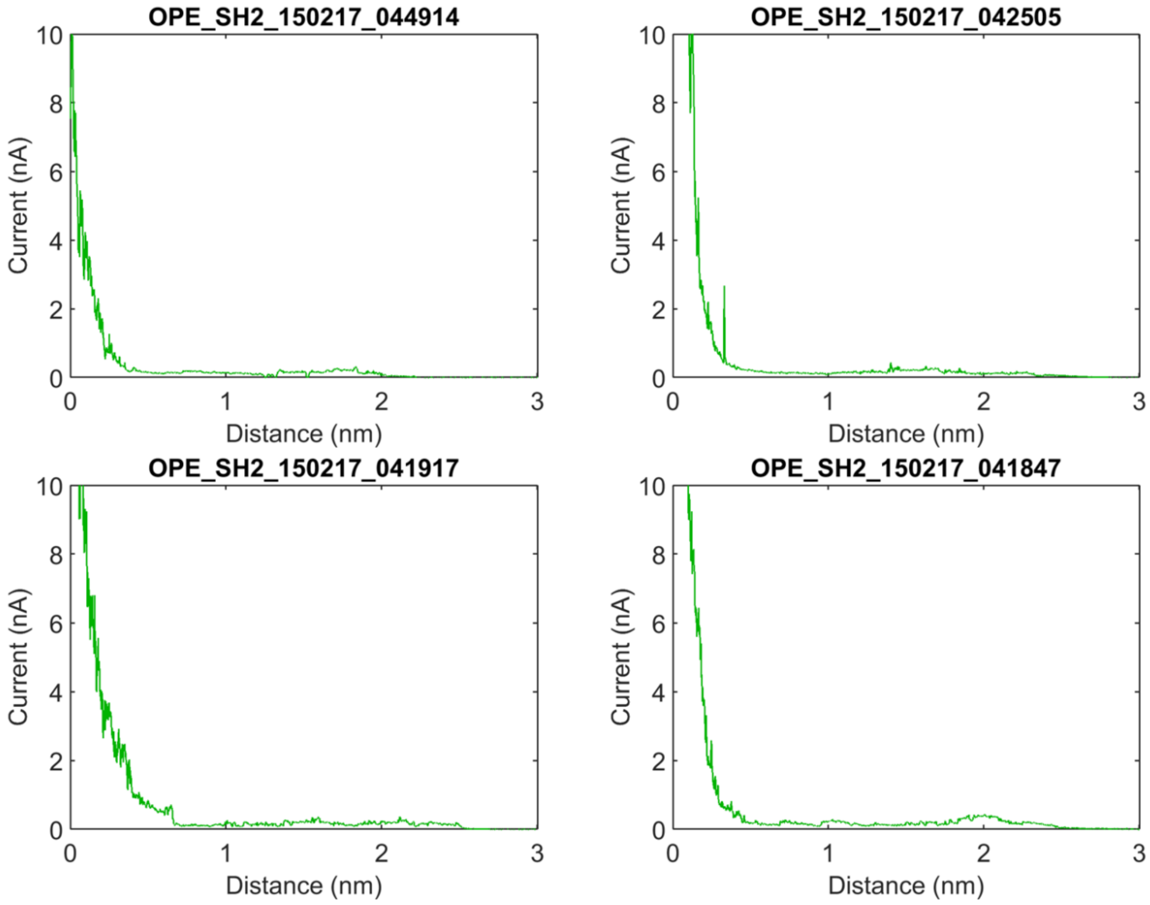

Supplementary Figure 18 - low *G* - OPE: Representative *I(s)* traces of the low conductance (green) group. We assume the low conductance state is due to the conduction through two coupled molecules.

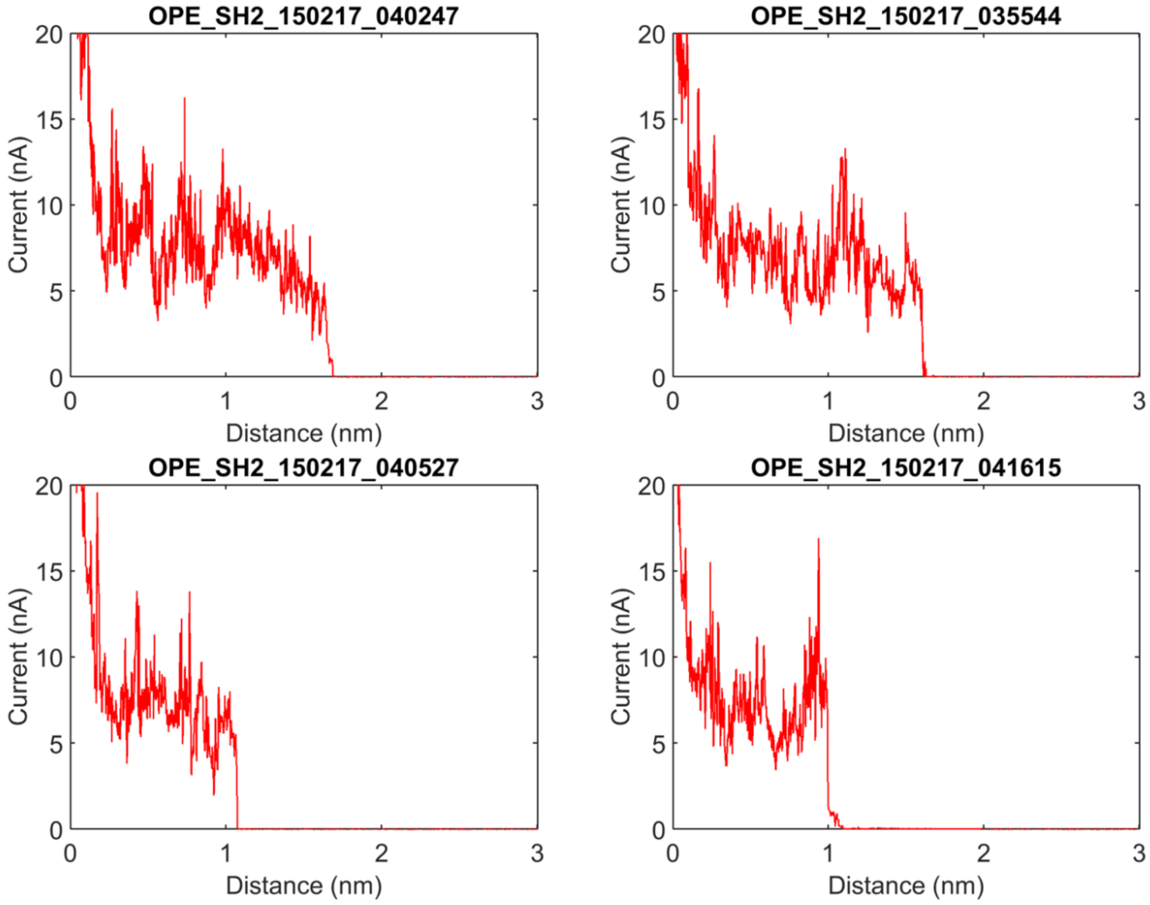

Supplementary Figure 19 - high *G* - OPE: Representative *I(s)* traces of the high conductance (red) group.

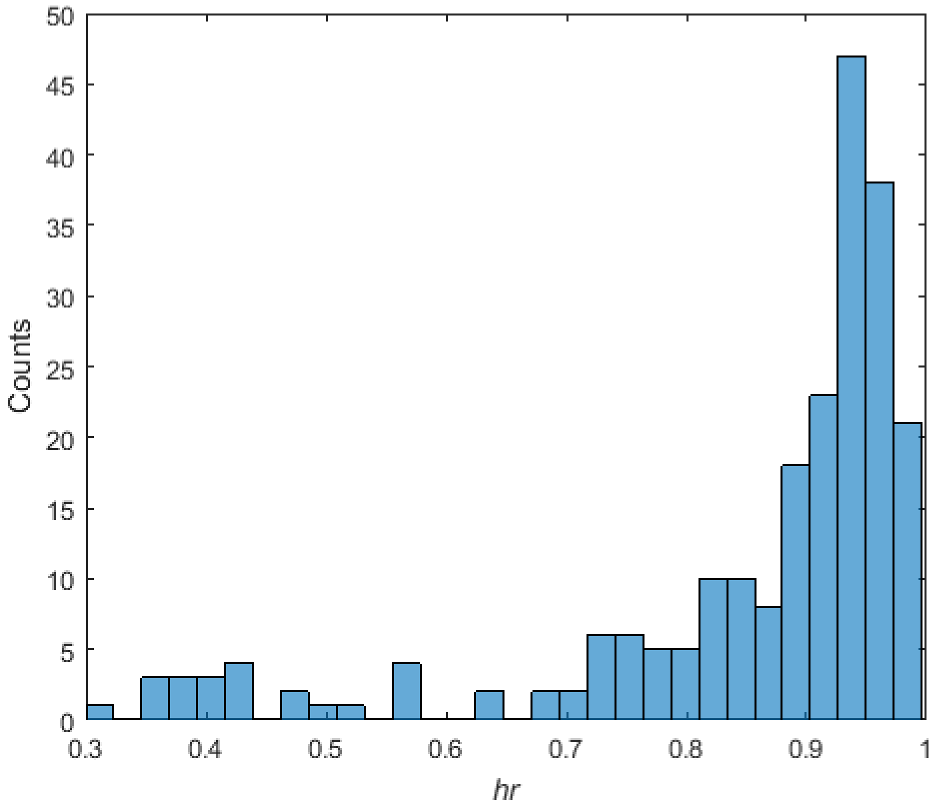

Supplementary Figure 20– OPE - low G: h_r histogram of the low conductance cluster of OPE. Plotting the histogram of the h_r indicates that there is a sub-population in the low conductance cluster towards low h_r (traces with h_r close to 1 feature short plateaus and are more similar to exponential decays) Bind width: 0.0232

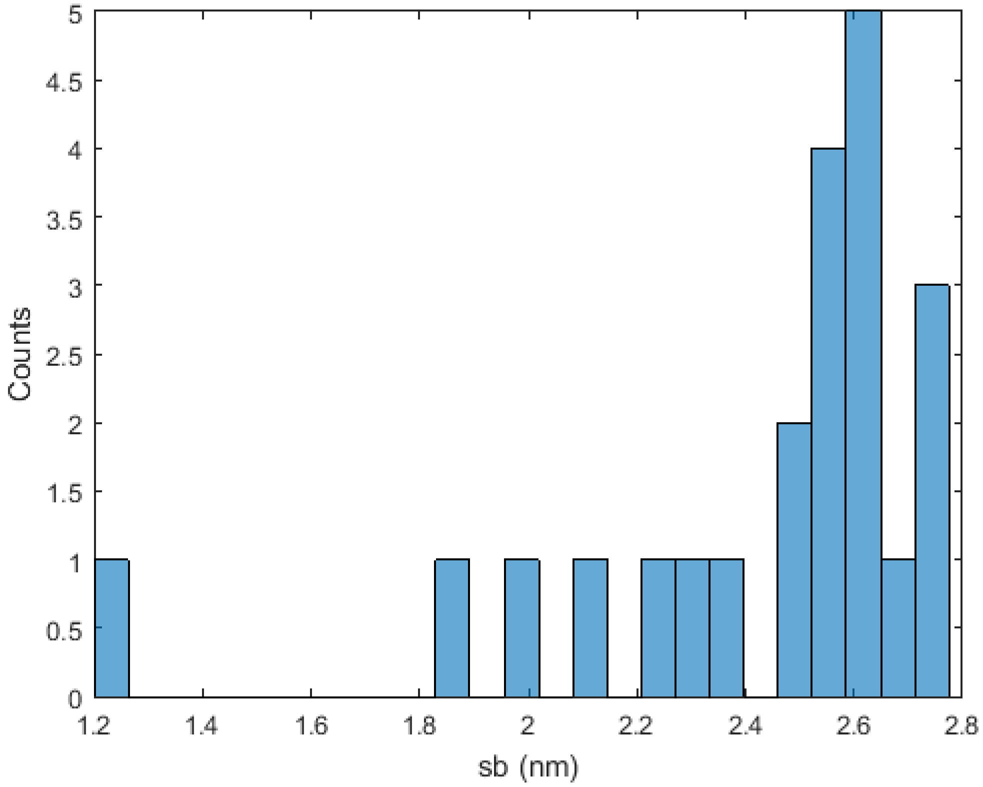

Supplementary Figure 21 –OPE - break-off distance low *G*, small h_r: Histogram of the break-off distance of the low h_r sub-population (h_r below 0.6) in the low *G* cluster. Plotting the s_b histogram of this sub-population of the low *G* cluster gives a most probable s_b with similar magnitude reported before for the two molecular conductance state. Bin width 0.026 nm.

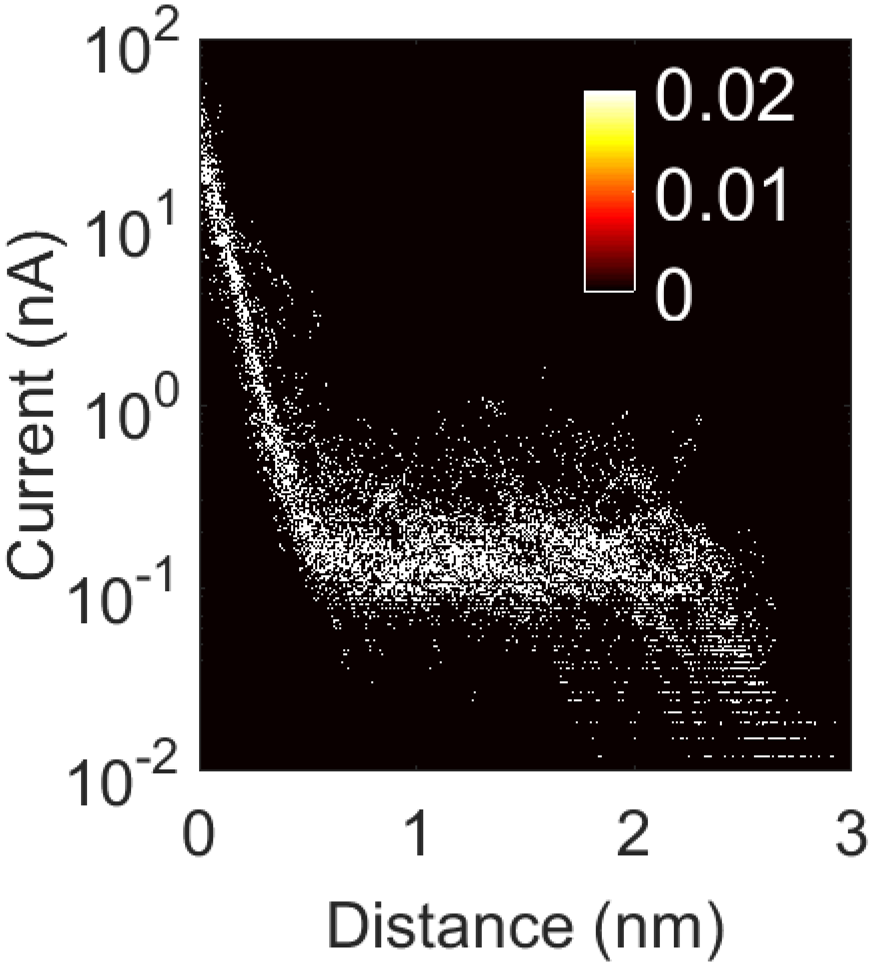

Supplementary Figure 22 – OPE - 2D log current histogram: Green, low *G*, cluster without high h_r traces. Current binning: 150 bins/decade, distance bin width: 0.004 nm

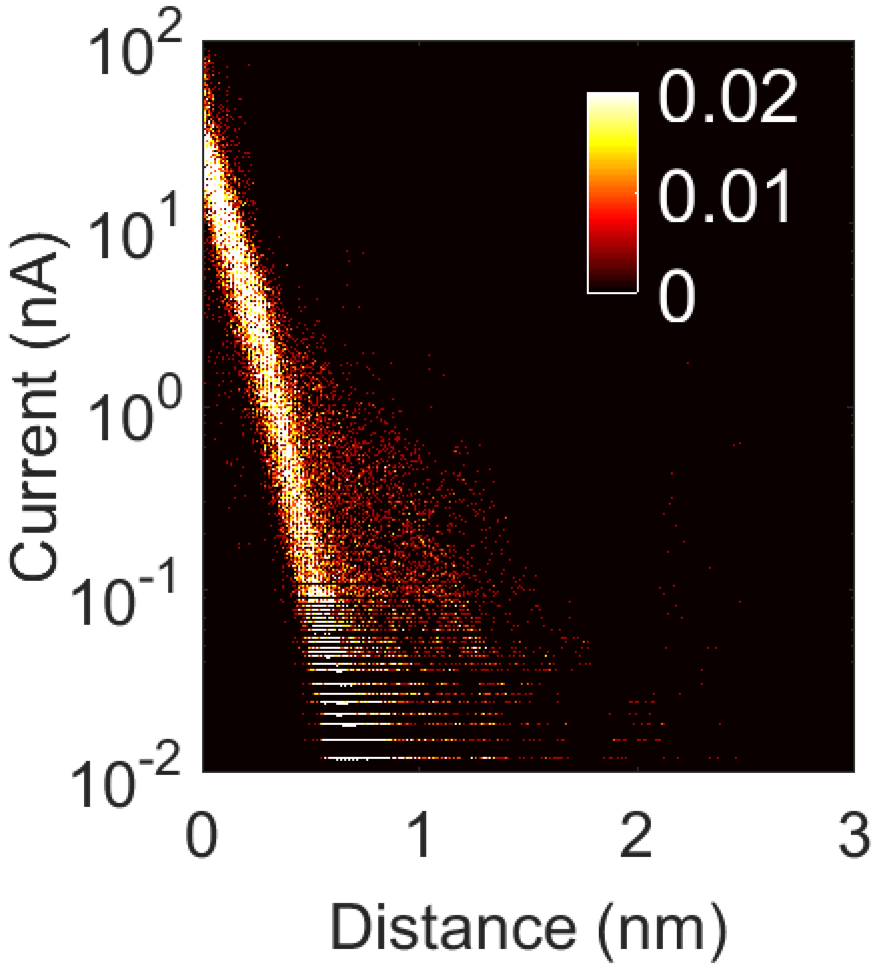

Supplementary Figure 23 – OPE - 2D log histogram: Green, low *G*, cluster excluding the low h_r graphs. Current binning: 150 bins/decade, distance bin width: 0.004 nm

Supplementary Note 1

Multiple Molecules in Junction

As discussed in the main text, it has been established in the past that there is the possibility of more than one molecule bridging a junction. It is assumed in this case that the measured plateau current is an integer multiple of the single molecular plateau current $k \cdot I_p$, corresponding to the number of molecules k bridging the gap. We investigated the effect on the (MPVC) by creating *I(s)* traces with conductance plateaus at integer multiples of I_p. To account for the junction formation probability (JFP), the probability of creating a junction with k molecules (i.e. higher conductance) was assumed to be $(\mathrm{JFP})^k$.

Simulated data sets contained 1000 *I(s)* traces each. 80 % of those traces were plain exponentials decays without plateau feature (Supplementary Figure 2 A to C, blue cluster) and 20 % with plateaus (i.e. JFP(k=1) = 20 %). Thus, 160 *I(s)* traces contained plateaus at I_p (red cluster), 32 plateaus at $2 \cdot I_p$ (green cluster) and 8 at $3 \cdot I_p$ (magenta cluster). The noise STDEV in Supplementary Figure 2 A was 0.1 nA. A STDEV of 0.1 nA was introduced to illustrate the influence of variations in the plateau current on separability with the MPVC. (Supplementary Figure 2 B) In C, the noise STDEV was increased from 0.1 nA to 1 nA to illustrate cluster formation at high noise levels.

In the low noise data set, panel A, the separation with MPVC is straight forward with *ΔX*. Both the plain exponential cluster and the clusters corresponding to multiple junction formation can easily be separated. Upon introduction of a STDEV to $I_{p,}$ the clusters elongate. Still, however, separation is straight forward. When a high noise STDEV is introduced (panel C), the clusters spread out. The $1 \cdot I_p$ and the plain exponential cluster start overlapping, but can still be separated using h_r, as demonstrated in the main text. In all three cases multiple molecules in the junction can clearly be isolated using the MPVC approach.

Supplementary Note 2:

MPVC with Density Clustering

Clusters with highly irregular shapes can be challenging to isolate with histogram based methods. It can be beneficial to use density based clustering in those cases. The data in Supplementary Figure 2 B was analysed with the density based clustering approach described by Rodriguez and Laio.[1] ΔX and θ were transformed into Cartesian coordinates and the pairwise distance between all points was calculated and input in the algorithm. The decision graph and the resulting clusters are shown in Supplementary Figure 3.

Using this density based clustering algorithm, all data points are correctly assigned to their cluster.

The disadvantage of using the density based clustering algorithm is, that a manual selection needs to be taken in the decision graph. The advantage is that the number of clusters is given.

Supplementary Note 3

Cluster number

In the manuscript, the Gustafson-Kessel fuzzy c-means (GK FCM) algorithm was used to cluster data. The GK FCM is an adaptation of the Fuzzy c-means algorithm that includes a covariance matrix, to allow for the clustering of advanced cluster shapes. It iteratively optimizes a given cost function to optimally assign observations to different clusters.[2,3,4,5] One of the downsides of the GK FCM, however, is that the number of clusters must be selected manually. Often with unlabelled data in machine learning algorithms the number of clusters is ambiguous and cannot be calculated exactly, so one needs to rely on visual confirmation or semi-accurate methods like the “elbow-method”, where the cost is plotted as a function of the cluster numbers (Supplementary Figure 5).

Supplementary Note 4

Reference Vector and Limitations

The choice of the reference vector influences the position and to some extend shape of the data in the polar representation. To illustrate this point, a simulated data set was generated with 1000 graphs, 20 % of which contained plateaus (Supplementary Figure 1, green data). The break-off distance of the plateaus was varied with a STDEV of 1 nm, leading to a long narrow cluster in the polar plot. The data set was subsequently analysed using three different reference vectors with exponential decay coefficients of 0.5 $Å^{-1}$, 1 $Å^{-1}$ and 5 $Å^{-1}$ (dark to light green in Supplementary Figure 1). The nature of the distribution does not change significantly in that the plain exponential cluster is at the base of the distribution (blue).

To point out limitations of the method, a data set was generated consisting of 3000 graphs, 1000 each containing plateaus at 0.5 nA, 2.5 nA and 5 nA with a s_b STDEV of 1 nm (Supplementary Figure 12). Plateaus with a wide range of currents and break-off distances lead to long, narrow, curved clusters. The clusters, although fairly obvious to the human eye, bring histogram based clustering methods and *k*-means clustering methods to their limits. In these cases density based methods might be advantageous.

Supplementary references

1. Rodriguez, A. & Laio, A. Clustering by fast search and find of density peaks. *Science* **344,** 1492–1496 (2014).
2. MacQueen, J. B. Some Methods for classification and Analysis of Multivariate Observations. *5th Berkeley Symp. Math. Stat. Probab. 1967* **1,** 281–297 (1967).
3. Kaufman, L. & Rousseeuw, P. J. Clustering by means of medoids. *Stat. Data Anal. Based L 1-Norm Relat. Methods. First Int. Conf.* 1–12 (1987).
4. Gustafson, D. E. & Kessel, W. C. Fuzzy Clustering with a Fuzzy Covariance Matrix. *IEEE CDC, San Diego, Calif.* 761–766 (1978).
5. Yang, M. S. A survey of fuzzy clustering. *Math. Comput. Model.* **18,** 1–16 (1993).
